# American literature news narration based on computer web technology

**DOI:** 10.1371/journal.pone.0292446

**Published:** 2023-10-16

**Authors:** Juan Liu, Sha Mi

**Affiliations:** College of Arts and Sciences, Northeast Agricultural University, Harbin, China; East China Normal University, CHINA

## Abstract

Driven by internet technology, online has become the main way of news dissemination, but redundant information such as navigation bars and advertisements affects people’s access to news content. The research aims to enable users to obtain pure news content from redundant web information. Firstly, based on the narrative characteristics of literary news, the Term Frequency-Inverse Document Frequency (TF-IDF) algorithm is employed to extract pure news content from the analyzed web pages. The algorithm uses keyword matching, text analysis, and semantic processing to determine news content’s boundaries and key information. Secondly, the news text classification algorithm (support vector machine, K-nearest neighbor, AdaBoost algorithm) is selected through comparative experiments. The news extraction system based on keyword feature and extended Document Object Model (DOM) tree is constructed. DOM technology analyzes web page structure and extracts key elements and information. Finally, the research can get their narrative characteristics by studying the narrative sequence and structure of 15 American literary news reports. The results reveal that the most used narrative sequence in American literary news is sequence and flashback. The narrative duration is dominated by the victory rate and outline, supplemented by scenes and pauses. In addition, 53.3% of the narrative structures used in literary news are time-connected. This narrative structure can help reporters have a clear conceptual structure when writing, help readers quickly grasp and understand the context of the event and the life course of the protagonists in the report, and increase the report’s readability. This research on the narrative characteristics of American literature news can provide media practitioners with a reference on news narrative techniques and strategies.

## 1. Introduction

With the rapid development of the internet and Hyper Text Markup Language (HTML) technology, news media has achieved the goal of faster news dissemination and richer news content through the characteristics of fast internet transmission. Svetlana Alexandravna Alexievich (Belarusian: Святлана Аляксандраўна Алексіевіч), a famous journalist, won the Nobel Prize for Literature in 2015. After that, literary news, which takes deep excavation as its responsibility, is increasingly recognized by society through displaying its value [1]. The traditional way of news dissemination has already transformed the direction of network news. The news media, taking advantage of the rapid transmission speed of the network, has given users a new experience in viewing, listening, and feeling [2]. The number of Internet users has increased dramatically, making the Internet the largest information base and the Web the largest information source. News web pages often inundated with excessive and unnecessary information. In addition to the main content, most web pages contain navigation bars, advertisements, copyright statements, and other unrelated information, referred to as noise [3], are commonly found. In the face of these pages mixed with redundant information, fast and accurate extraction of news text data plays a vital role in text mining, news reporting, and public opinion guidance in the later stage.

Extensive research has been conducted by scholars in the field of web page text-mining technology, albeit with varying objectives and research focuses. Liu et al. used the web crawler developed by Python to collect Ctrip and TripAdvisor’s visitor comments on the "Macao attractions" theme. They investigated the changes in Macao’s tourism image from 2014 to 2018 based on user-generated content on tourism websites [4]. Text mining was used to analyze the content of the two largest tourism websites, revealing the online destination image of Macao. Hudaefi et al. took Indonesia as the research object and conducted qualitative research through text mining. During the Corona Virus Disease 2019 (COVID-19) epidemic, they found the Indonesian government’s assistance mode in the management of zakat. This discovery contributed to enhancing readers’ and scholars’ understanding of zakat management within the context of the epidemic [5]. This research was conducive to understanding zakat’s contribution to managing COVID-19. Koseoglu et al. proposed an intelligence and analysis model that could be used to analyze competitors. Text mining technology was combined with network analysis as a big data method. The survey results demonstrated that online reviews might be a reliable source of information [6]. The competitor analysis method proposed can be used in the hotel industry and many other industries. The above research used text mining technology to explore the image of scenic spots, government management, competitor analysis, and other aspects, indicating the advantages of text-mining technology. However, few studies employed text mining and analysis to analyze literary news.

For the study of online news text extraction and news classification, Reis et al. analyzed the main characteristics of fake news detection. They measured the predictive performance of current methods and features of automatic detection of fake news [7]. Liu et al. proposed a new Deep Neural Network (DNN) to detect fake news early, and the results revealed that the method only needed 10% of labeled fake news samples to achieve this effect in the PU learning setting [8]. Nasir et al. combined convolutional and recurrent neural networks for fake news classification. The model was successfully validated on two fake news datasets (ISO and FA-KES) and obtained significantly better detection results than other non-mixed baseline methods [9]. Jang et al. explored the performance of word2vec convolutional neural networks (CNNs) for classifying news articles and tweets into related and unrelated articles. Experiments denoted that CNNs using the word2vec model were more effective on news articles than tweets because news articles are generally more uniform [10]. Jwa et al. investigated data-driven automated detection of fake news. They found that the deeply contextualized nature of Bidirectional Encoder Representations from Transformers (BERT) was best suited for this task and improved the F1 score by 0.14 over older, state-of-the-art models [11].

The above research made some progress in the text extraction and news classification of network news, but there were still some limitations and problems to be solved. For example, although Reis et al. evaluated the predictive performance of automated methods for detecting fake news, other unaccounted factors may influence the results. Liu et al. ’s approach presented promising results in PU learning settings, but more research may be needed to verify its generality to other datasets and real-world applications. Nasir et al. ’s model performed well in classifying fake news, but the limitations of the data set and specific tasks may have limited the study. Jang et al. found that word2vec CNNs were more effective on news articles, but this conclusion may not apply to other types of text data. Finally, the study by Jwa et al. demonstrated the advantages of the BERT model for fake news detection, but further evaluation of its robustness under different environments and data sets was still needed. Hence, based on the contributions and shortcomings of the above previous studies, given the large amount of redundant information (such as navigation bars, advertisements, etc.) in the current network news pages, the information affects users’ access to news content. Therefore, this research aims to use computer network technology, especially based on the Term Frequency-Inverse Document Frequency (TF-IDF) algorithm, to extract pure news content from the analyzed web pages, thus meeting the needs of users for pure news information. Besides, previous research has shown that spreading and detecting fake news is an important issue. Reis et al. analyzed the main features of fake news detection; Liu et al. proposed a DNN for early detection of fake news; Nasir et al. successfully classified fake news by combining convolutional and recurrent neural networks. However, these studies focus on the whole news or specific types of news, and lack attention to the narrative characteristics and structure of American literary news. Consequently, the purpose of this research is to further explore the narrative characteristics of American literary news through the analysis of its narrative order and structure, and to provide a reference for media practitioners of news narrative skills and strategies. To sum up, according to the above relevant studies, this research is of great necessity for solving the problem of redundant information in network news and exploring the narrative characteristics and structure of American literary news. By using computer network technology to extract pure news content and study the narrative characteristics of American literary news, it can provide a better news browsing experience for users and offer guidance for media practitioners on news narrative skills and strategies.

The research first starts with the narrative characteristics of literary news, and uses the Term Frequency-Inverse Document Frequency (TF-IDF) algorithm to extract pure news content from parsed web pages. The algorithm uses keyword matching, text analysis, and semantic processing techniques to determine the boundaries and key information of news content. Second, the classification algorithm of news text classification (support vector machine (SVM), K-nearest neighbor (KNN), AdaBoost algorithm) is selected through comparative experiments. This research builds a news extraction system based on keyword feature and extended Document Object Model (DOM) tree, which uses DOM technology to parse the web page structure and extract key elements and information. Lastly, the narrative characteristics of 15 American literary news reports are studied. The narrative structure used helps journalists have a clear conceptual structure when writing, helps readers quickly grasp and understand the background of events and the life trajectories of the main characters in the report, and improves the readability of the report. This research offers an in-depth discussion of the narrative characteristics of American literary news and provides a reference for media practitioners in news narrative techniques and strategies.

## 2. Materials and methods

### 2.1 Discussion on the narrative characteristics of literary news

Literary news, also known as narrative news and non-fiction writing, emerged in the early 20th century in the United States. In the 1960s, it ushered in a prosperous period in magazines such as Mr. Fashion, Rolling Stone, The New Yorker, and Harpers [12]. Literary news represents a multifaceted and contextual form of writing that adds allure to news reports. It incorporates various elements, involving in-depth investigation, accurate information, a focus on human emotions, and integration of new facts and literary writing [13]. News narration is produced through the continuous integration of news and narration. Its primary focus is on analyzing news reports, specifically examining the narrative principles and methods employed within them. News narration is the analysis of many news reports based on possessing detailed analysis materials, explaining the characteristics, methods, and functions of narrative, and concluding the general principles [14].

The narrative characteristics of literary news are commonly analyzed from the perspective of time, structure, and other aspects. The narrative is the perspective of observation and time, which is also the perspective of the text. Different narrative angles employed in depicting the same subject or individual yield distinct texts. Multiple perspectives are often used in news reports, which can be divided into omniscient editorial perspective, first-person witness narrative and third-person party alternate, indefinite party, and first-person party narrative [15]. Narrative time holds significant importance in narratology, which usually focuses on two factors: time sequence and duration. The time sequence refers to the relationship between the events in the story and the narrative text, which can be categorized into five types: sequential narration, flashback, pre-narration, interlude, and supplementary narration. News reports have three narrative ways: sequence, flashback, and pre-order. Duration is the relationship between the total time in the story event and the total time to narrate the event. It can be classified into five types: ellipsis, synopsis, scene, slowing down, and pause. Among them, ellipsis and synopsis accelerate the pace of the original story. The scene is about equal to the original story speed, and the pause is to delay the original story speed through "pause" [16]. The narrative structure encompasses the organizational form of content and the surface structure of text narration. Its focus is on organically arranging events, characters, and the environment. The narrative structure of literary news is divided into three types: time-connected narrative structure, cause-and-effect narrative structure, and cross-parallel narrative structure. The time-connected narrative structure pertains to the arrangement of events in the report based on their chronological or developmental sequence. Cause-and-effect narrative structure refers to the type that uses the logical relationship existing in the event to arrange and combine the material. The cross-parallel narrative structure uses the similarities between materials to carry out a parallel narrative [17].

### 2.2 Technical analysis of keyword extraction

In the context of web page information extraction, web crawlers are utilized to crawl web pages. The HTML source code is parsed into a Document Object Model (DOM) or DOM tree. Subsequently, the page is partitioned according to the DOM tree. Each block undergoes data feature calculations to extract relevant keywords. These keywords are employed to calculate the relevance between the content of the page block and its topic. An appropriate classification algorithm is selected to establish a classifier to distinguish content and noise. Finally, the extracted news is classified by text classification algorithm and stored in the database [18, 19]. Throughout this process, keyword extraction assumes a crucial role.


$${TF}_{a,b}=\frac{p_{a,b}}{\sum_{i}p_{i,b}}$$ (1)

In Eq (1), *p*_*a*,*b*_ indicates the frequency of words and sentences in the document. ∑_*i*_*p*_*i*,*b*_ represents the total number of all words in the document. The expression of IDF is:

$${IDF}_{a}=log\frac{\left|N\right|}{\left|\left\{b:q_{a}\in n_{b}\right\}\right|}$$ (2)

In Eq (2), |*N*| represents the total number of documents in the corpus. |{*b*: *q*_*a*_∈*n*_*b*_}| indicates the number of documents containing words. The calculation of the TF-IDF weighting method reads:

TF−IDF=TF*IDF (3)

Therefore, the words with higher frequency in the target document and lower frequency in the corpus will have greater weight [23].

### 2.3 Keyword-based content extraction algorithm

**Fig 1:**
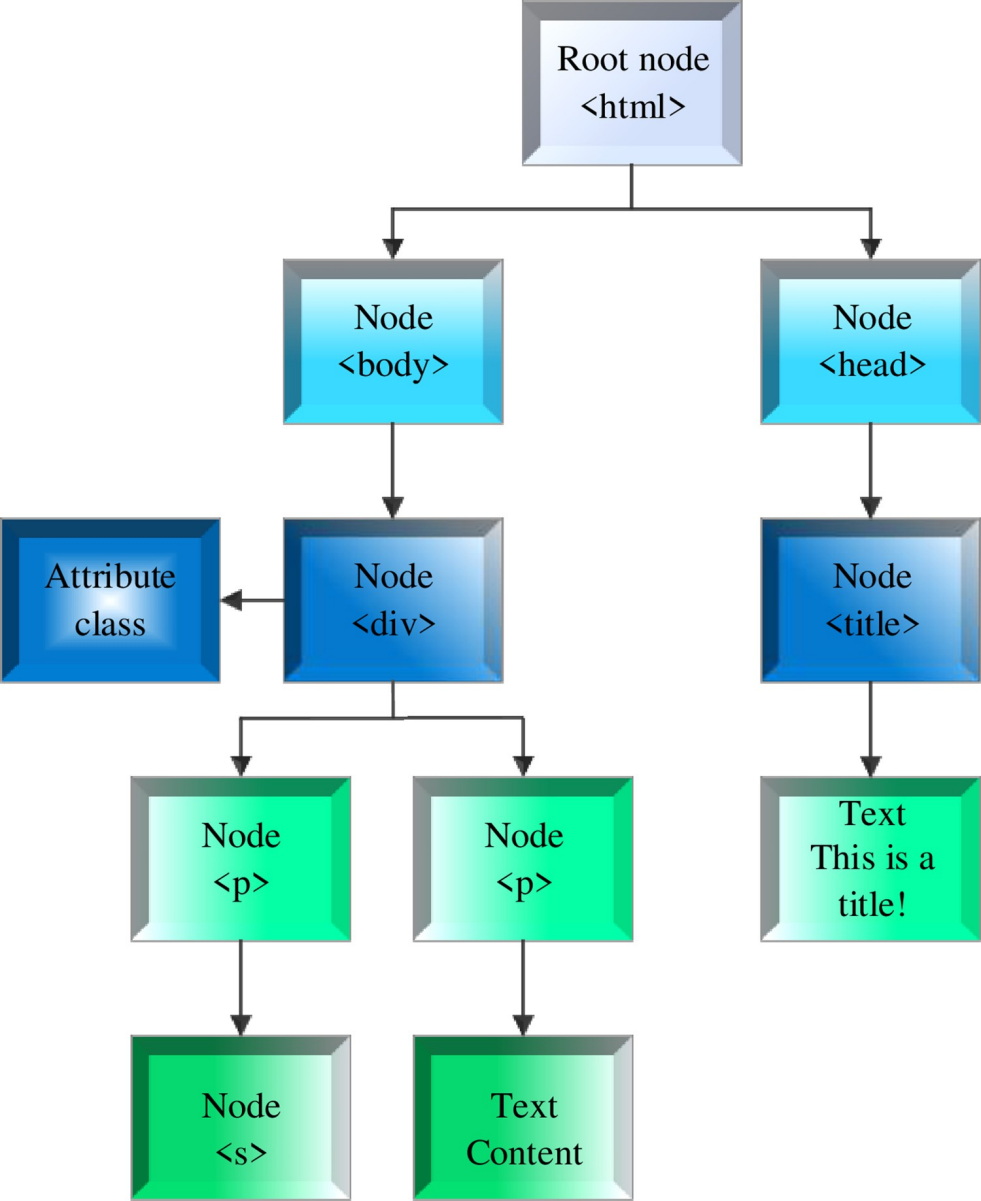
DOM tree.

The current web page structure is very complex, with many nodes. It will be time-consuming to calculate the eigenvalues of each node directly. Therefore, some nodes with noise that are easy to judge are removed first. Then, nodes are fused to form a web page block through label path similarity. These web page blocks form an extended DOM tree. DOM is a common technique for parsing and manipulating the web page structure. It can represent a web page document as a tree of nodes, each representing a different part of the web page (e.g., headings, paragraphs, links, etc.). By using DOM technology, researchers can access and extract specific elements in a web page, such as text content, tag attributes, and so on, and conduct further analysis and processing on them. This research chooses DOM technology to analyze the web page structure to obtain the data needed for the narrative characteristics of American literary news reports. By analyzing the web page structure, researchers can effectively extract and analyze key information such as text content, narrative time, and narrative structure of news reports, to deeply study these reports’ narrative characteristics and patterns. In a word, the purpose of using DOM to analyze web page structure is to acquire the data needed for the narrative characteristics of American literary news reports and to further explore the narrative ways and structural characteristics of these reports through the analysis of web page structure. The eigenvalue calculation process is carried out for each node on the extended DOM tree. A part of the DOM tree parsed from a web page is displayed in Fig 1 [24].

In Fig 1, taking the root node as the first layer, the three <p> tags in the fourth layer of the DOM tree will be fused into one. There are two situations when using the label path to block the web page. One is that all the text nodes have the same <div> father node, so the label path block method degenerates into the method of <div> block. The other is that the text block may contain multiple <div> blocks of the same level. Thus, the <p> nodes of the same level but in different <div> can be merged into the same block through the label path block method.

The most difficult part to identify in most news pages is the copyright statement at the end of the page. The structural style and text density closely resemble those of text nodes. If only the surface features are difficult to identify, it is necessary to introduce the feature of topic relevance [25]. TF-IDF algorithm is employed to extract keywords. Then, keywords are applied to calculate topic relevance. The proportion of the number of all title keywords in other text blocks is taken as the topic relevance C, as follows:

$$C=\frac{N_{wordK\cap wordT}}{N_{wordK}}$$ (4)

In Eq (4), *N*_*wordK*∩*wordT*_ refers to the number of title keywords in other text blocks. *N*_*wordK*_ indicates the number of keywords in the title [26]. In most web pages, besides the body of the page, there may also be a navigation bar, content recommendation, etc. To eliminate this part of the noise, each link node is calculated with a link text ratio and compared with the set threshold to identify the noise. The link text ratio calculated for each page block determines whether the link should be treated as a normal character or deleted. The calculation can be written as Eq (5):

$$L(y)=\frac{\sum_{j\in V_{y}}n_{j}}{\sum_{j\in V_{y}}m_{j}}$$ (5)

**Fig 2:**
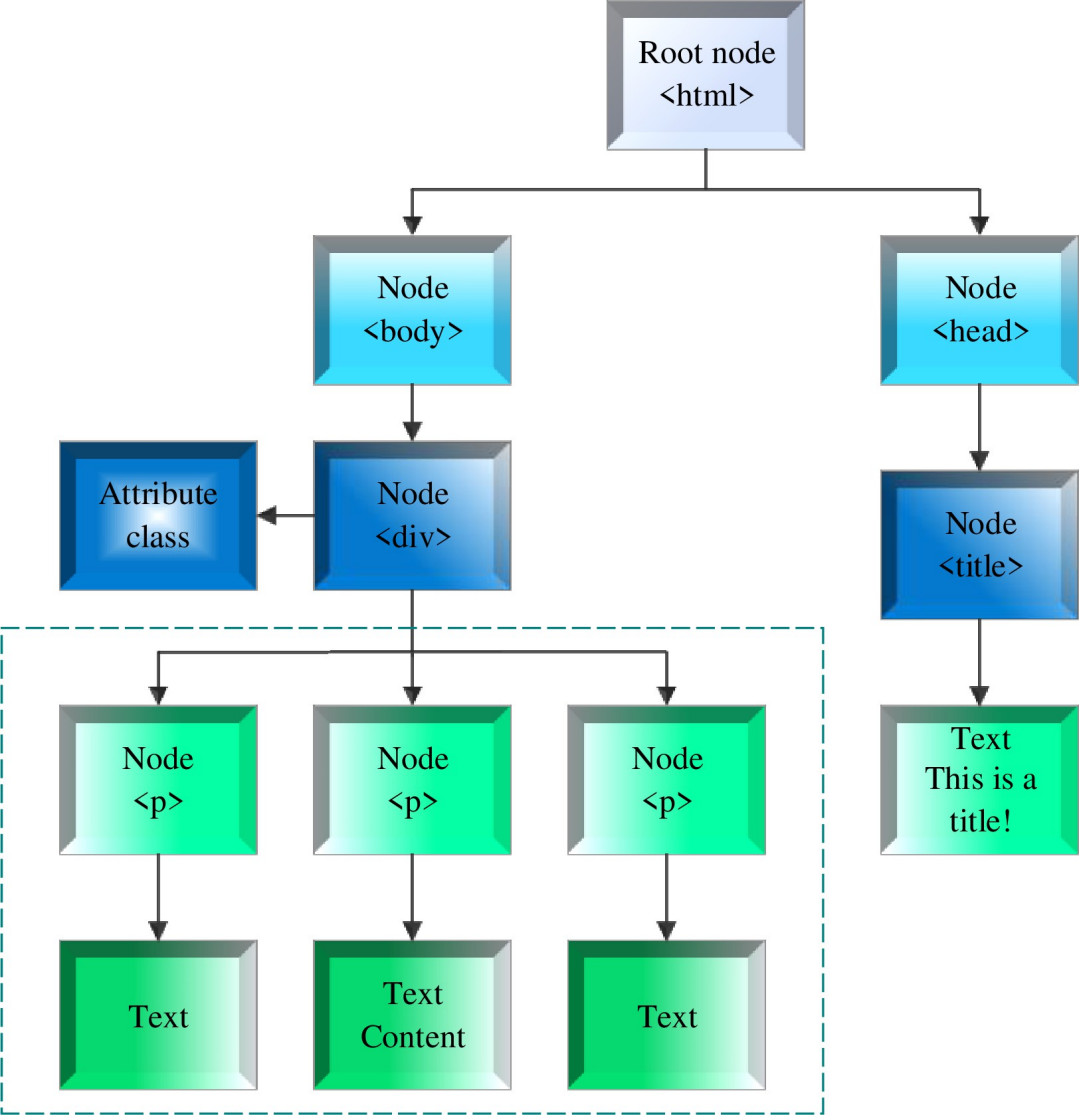
Extended DOM tree.

In Eq (5), y means the page block label path; *V*_*y*_ refers to the node collection accessible by path y; *n*_*j*_ and *m*_*j*_ represents the number of link characters and all characters in node j [27]. After pre-processing, the DOM tree in Fig 1 becomes an extension of Fig 2.

In Fig 2, the dotted part is the three nodes on the original DOM tree. Because the paths of tags are the same, they will merge into a node on the extended DOM tree. The <s> tag does not exist on the extended DOM tree. This web page is transformed into a plain text web page [28].

### 2.4 Classification process and evaluation method of the classification algorithm

**Fig 3:**
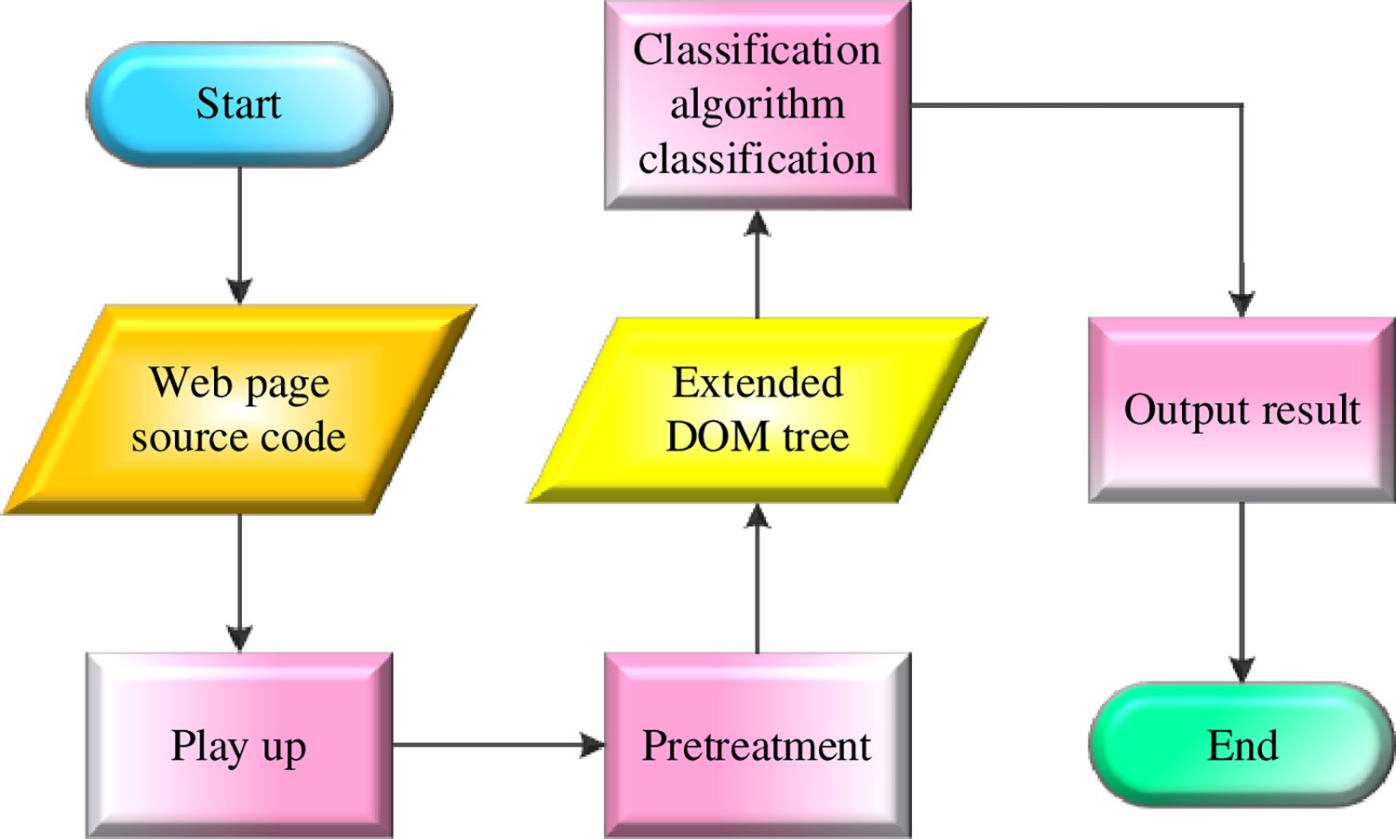
Classification process of new web page nodes by the classification model.

After extracting news using tag path and keyword features, an appropriate classification algorithm is selected to establish a classifier to distinguish content and noise. Commonly used text classification algorithms include SVM, KNN, AdaBoost algorithm, etc. SVM is one of the widely used binary classification models that define the classifier with the largest interval in the feature space. Its basic idea is to find a hyperplane in the feature space that maximizes the class interval [29]. KNN is a basic classification and regression method. In its classification algorithm, the KNN input is the feature vector of each instance, and the output is the corresponding category. Unlike the SVM model, the KNN algorithm has no learning process. Its basic idea is to vote with the K data closest to the data of the unknown class according to the existing labeled data. Most of the classes in the voting result are those of unknown instances [30]. The AdaBoost algorithm, in contrast to individual classifiers like SVM and KNN, is an ensemble method that combines multiple weak classifiers to form a strong classifier. Its basic idea is to synthesize the judgment results of multiple weak classifiers on a task to get a result that will be better than the results of any weak classifier [31]. The classification model classifies the nodes on the DOM tree of the new web page extension, as plotted in Fig 3.

In Fig 3, a web page with text content is parsed into a DOM tree after obtaining the source code of the web page rendered by the browser. Then, nodes are fused according to the fusion rules to generate an extended DOM tree. After that, the nodes on the extended DOM tree are calculated with four eigenvalues. Following the classification by the classifier model, the nodes are assigned their respective categories [32, 33].

Evaluation of classification methods commonly employs precision, recall, accuracy, and F1 value [34]. It can be assumed that R and N represent that the positive prediction result is correct and incorrect. F and T indicate that the negative prediction result is wrong and correct. Accuracy is the proportion of correctly predicted data in all test data, as shown in Eq (6):

$$Acc=\frac{R+T}{R+T+F+N}$$ (6)

Recall means the proportion of correctly predicted data in all positive test data, its expression is as follows:

$$Rec=\frac{R}{R+N}$$ (7)

Precision refers to the proportion of correctly predicted data in the positive data, as illustrated in Eq (8):

$$Pre=\frac{R}{R+F}$$ (8)

F1 value is the harmonic average of precision and recall, taking into account both precision and recall, as expressed in Eq (9):

$$F1=2*\frac{Pre*Rec}{Pre+Rec}$$ (9)

The precision and recall are between 0 and 1. The closer this rate is to 1, the better the classification effect of the model is.

The Normalized Discounted Cumulative Gain (NDCG) is used to evaluate the quality of ranked lists or recommended results. The NDCG is an indicator that is evaluated based on the quality of the ranking results. It considers each item’s ranking order and relevance, discounting the relevance and accumulating scores. By normalizing the cumulative score, the NDCG can provide a score between 0 and 1 that measures the quality of the ranked list. A higher NDCG score indicates a higher quality of ranking lists or recommendation results, which can better meet user needs. The calculation expression of NDCG reads:

NDCG@k=DCG@k/IDCG@k (10)

$$DCG@k={rel}_{1}+\frac{{rel}_{2}}{\log_{2}2}+\frac{{rel}_{3}}{\log_{2}3}+\cdots +\frac{{rel}_{k}}{\log_{2}k}$$ (11)

$$IDCG@k={rel}_{1^{*}}+\frac{{rel}_{2^{*}}}{\log_{2}2}+\frac{{rel}_{3^{*}}}{\log_{2}3}+\cdots +\frac{{rel}_{k^{*}}}{\log_{2}k}$$ (12)

NDCG@k and Discounted Cumulative Gain (DCG) @k is the NDCG value and cumulative discount gain calculated on the first k items or results. Ideal Discounted Cumulative Gain (IDCG) @k is the cumulative discount gain calculated on the first k items or results under ideal conditions. *rel*_*i*_ shows the relevance of the i-th item in the ranking list; *rel*_i*_ refers to the relevance of the i-th item in the ranking list in an ideal situation, namely, in descending order of relevance.

### 2.5 News extraction system based on keyword feature and extended DOM tree

**Fig 4:**
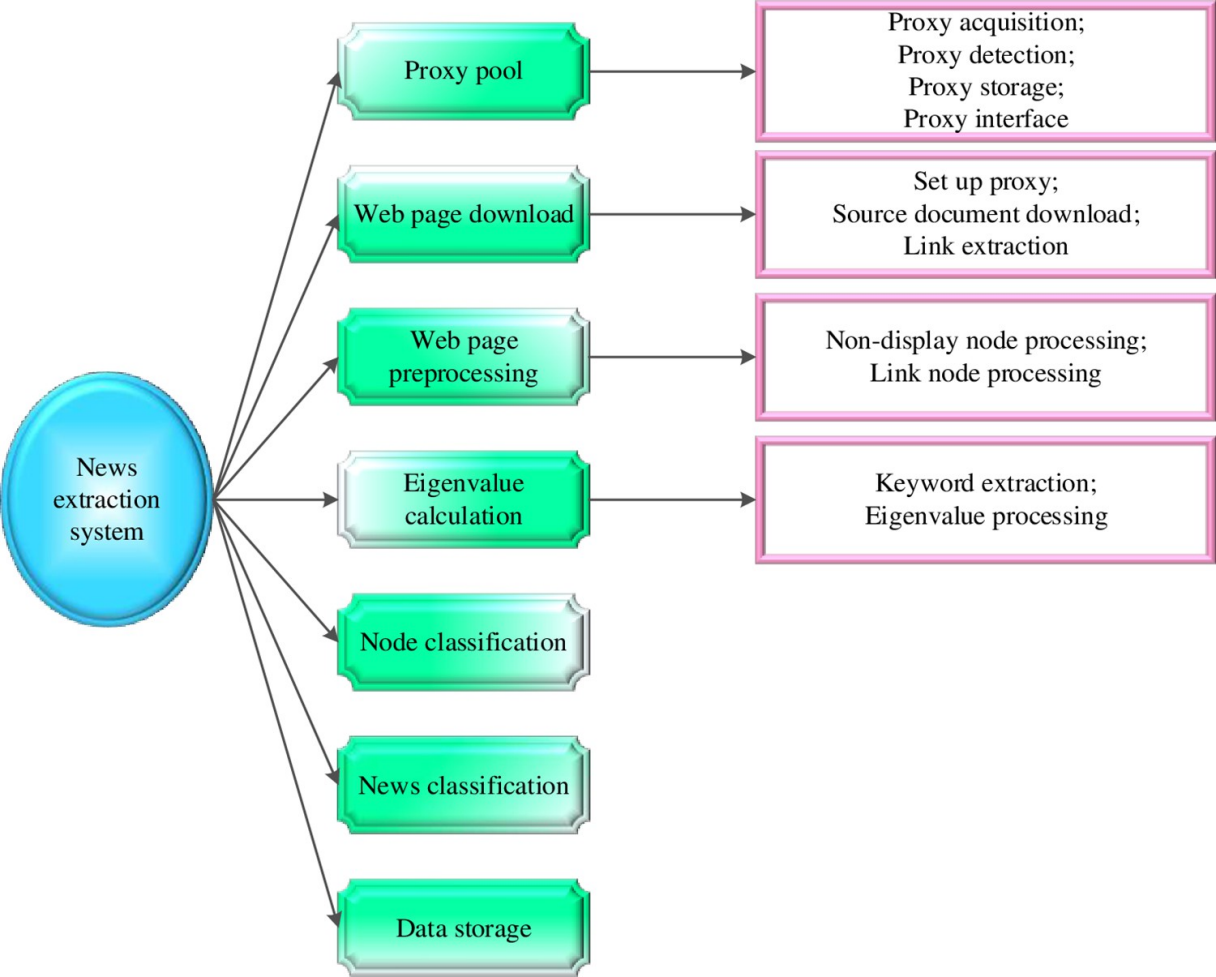
News extraction system based on keyword feature and extended DOM tree.

The news extraction system is constructed based on the above keyword features and the extended DOM tree algorithm. This research opens a web page with Chrome DevTools and switches to the Elements tab to view the DOM structure of the web page. The system structure is suggested in Fig 4.

In Fig 4, the entire news extraction system constitutes the first layer, encompassing several subsystems, such as proxy pool web page download, web page pre-processing, etc. These subsystems are the second layer, further dividing certain subsystems into more specific functions. The agent pool system comprises acquisition, detection, storage, and interface modules. There are also proxy settings, source document downloads, and link extraction modules under the web page download system. The web page pre-processing system is divided into non-display node processing, link node processing, and image node processing modules. The feature value calculation system, constituting the third layer, consists of keyword extraction and feature value processing modules. The first layer implements the corresponding functions by calling the second layer system. The second layer completes the corresponding operation by calling the module of the third layer [35, 36]. Consequently, each module performs a single function, proper size, high cohesion, and low coupling between modules.

### 2.6 Experimental environment and configuration

The experimental environment and parameter settings used in this experiment are exhibited in Table 1.

**Table 1:** The experimental environment and parameter settings.

Experimental environment/parameters	The setting of environment/parameters
Operating system	Windows 10
Processor	AMD 3600 @3.6Hz
Memory	16GB
Hard disk	1TB
Programming environment	Python 3.7
Parameter adjustment method	Grid Search CV
Machine learning library	Scikit-learn

This research selects 15 literary news reports from the American Fashion magazine "Mr. Fashion" for sample analysis. Since its establishment in 1933, the magazine has been the main media platform for publishing American literary news. These reports have a certain influence and representation in the field of literary news. Firstly, by analyzing these reports, important insights about literary news narratives can be gleaned from them. Secondly, based on the information provided, the subject mainly analyzes the sample from two narrative time and structure aspects. Narrative time and structure focus on the narrative timeline and how narrative structure is organized in literary news reporting. Analyzing these aspects reveals the pattern and trend of narrative features in literary news reports. Finally, on the basis of the analysis of these narrative characteristics, this research deeply understands the narrative mode and structural characteristics of American literary news reports. It reveals the influence of narrative mode in literary news reports on transmitting information, arousing readers’ interest, and shaping narrative angles and viewpoints.

## 3. Results and discussions

### 3.1 Comparison of results of different classification algorithms

**Fig 5:**
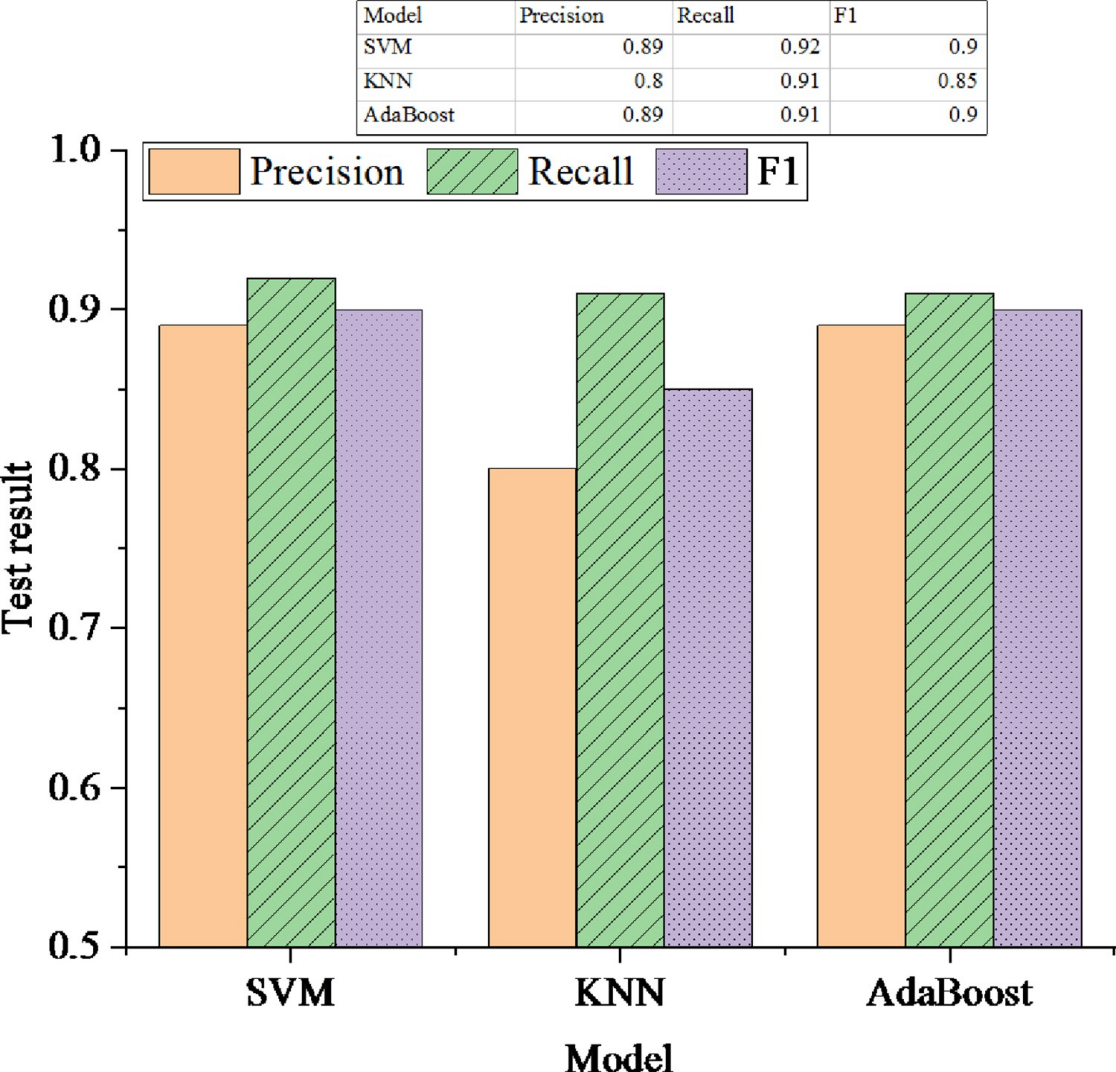
Test results of the classification algorithm.

The comparison experiments are used to determine the choice of the classification algorithm. The experimental data comes from the captured enterprise news page. The specific news page’s source code follows the rules of the target website. The total number of node data captured by the web crawler is about 25000. The 5-fold cross-validation method is used to compare the dataset’s three classification algorithms, as revealed in Fig 5.

Fig 5 denotes that the KNN algorithm is not as good as the other two algorithms regarding precision and recall. The precision of SVM and AdaBoost algorithms is the same as that of F1, 0.89, and 0.90. Nevertheless, the recall of SVM is 0.92, slightly higher than the 0.91 of the AdaBoost algorithm. Therefore, this experiment selects SVM as the node classification algorithm of the news extraction system.

### 3.2 Analysis of narrative time

**Fig 6:**
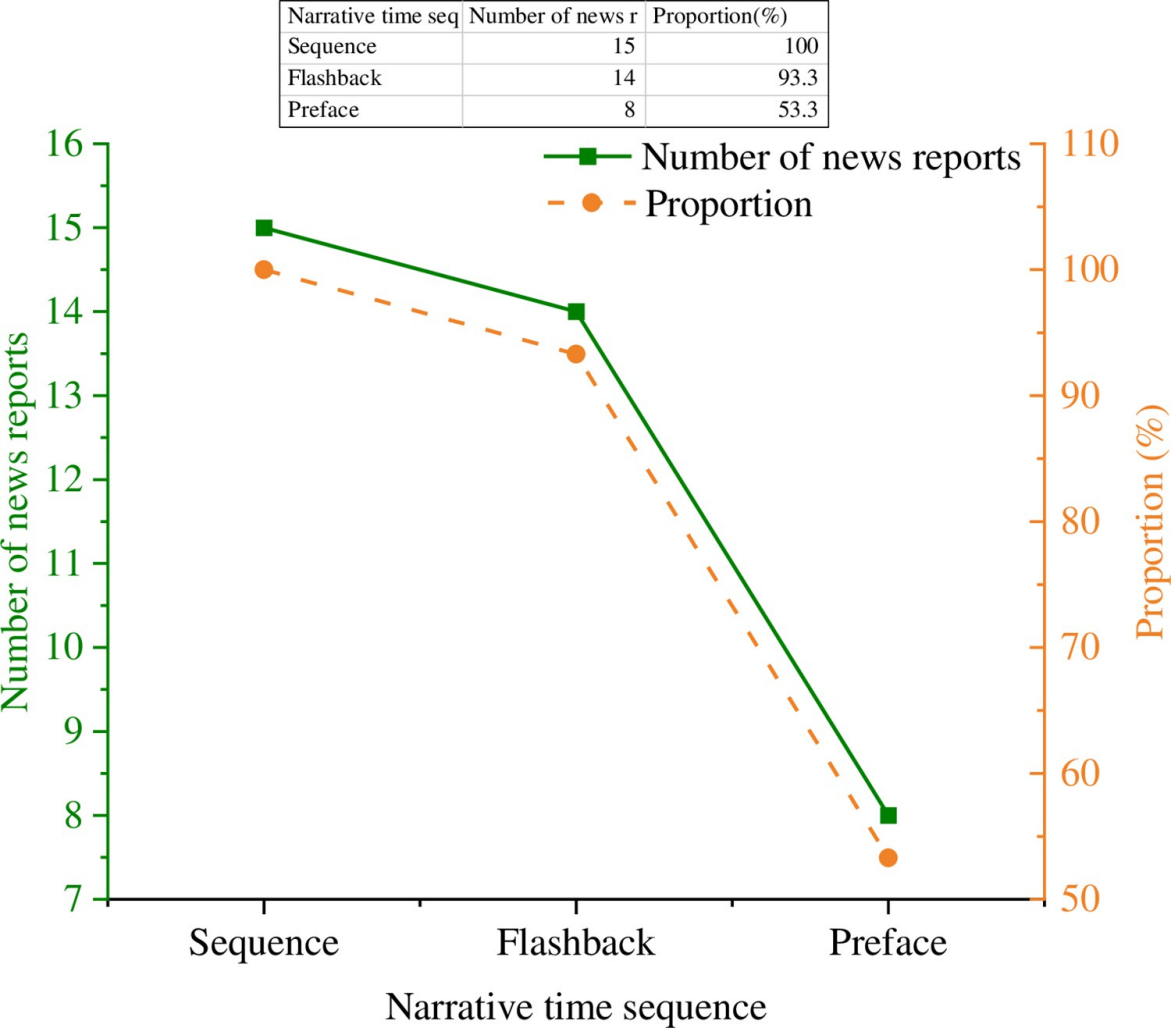
Analysis results of narrative time.

Narrative time is divided into two factors: time sequence and duration. The results of the time series analysis of 15 samples are signified in Fig 6.

In Fig 6, sequence and flashback are the most used narrative sequences in literary news. Most literary news uses these two-timing methods. In addition, literary news will not use only one narrative sequence but at least two or more narrative sequences. The experiment signifies that the narrator uses multiple narrative sequences in each report section.

**Fig 7:**
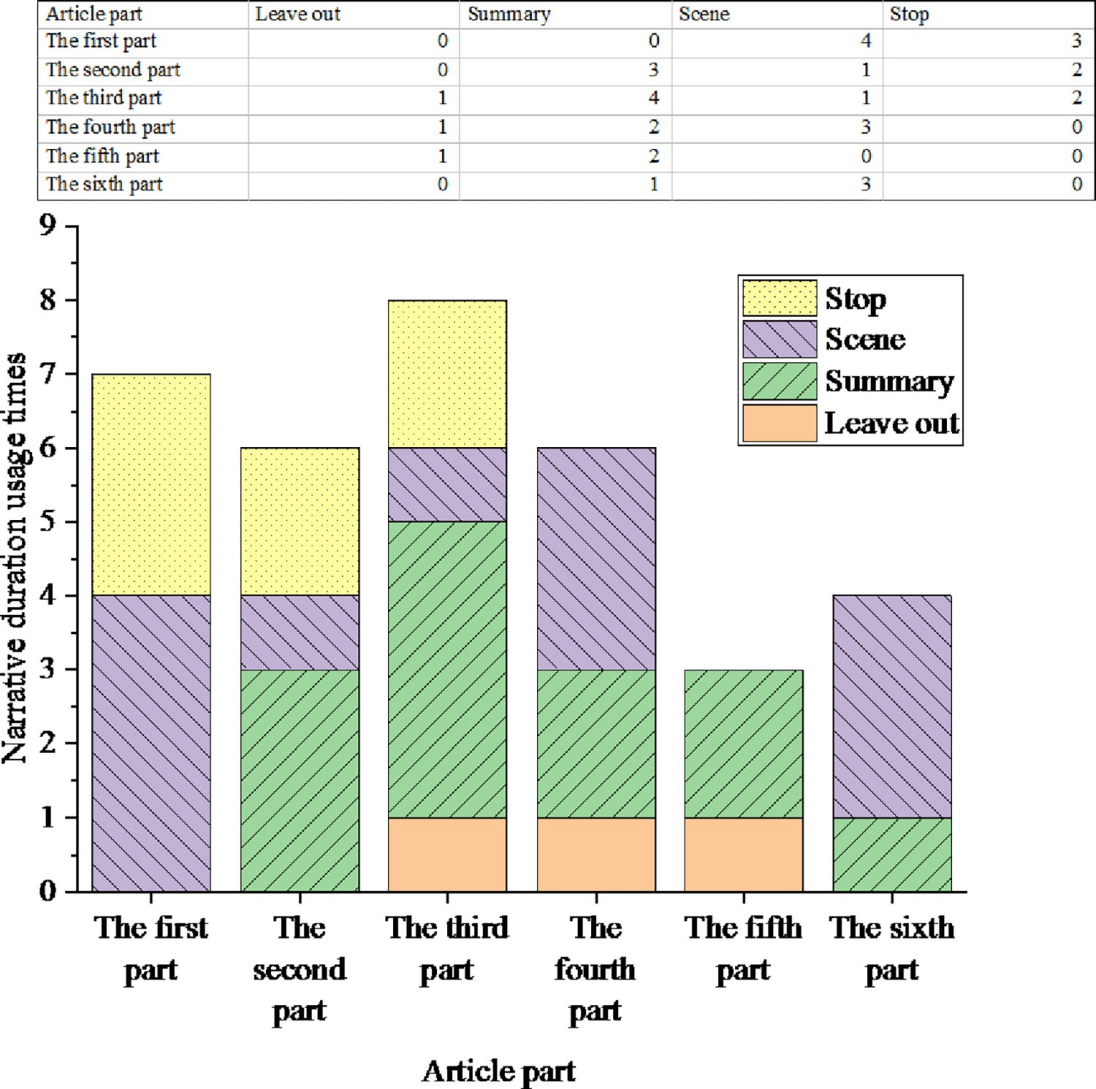
Analysis results of narrative duration.

Narrative time is employed to analyze journalist Tom Judenow’s Why We Should Applaud Him. This report is over 10,000 words long and divided into six parts. The analysis result of time length is demonstrated in Fig 7.

Fig 7 reveals that this report employs 12 scene narratives, 12 rough narratives, 3 omitted narratives, and 7 pause narratives. Additionally, there are a total of 15 instances of summary and ellipsis. The most frequent use is in narrative duration, followed by scene narration, and finally by pause narration. Because literary news reports should be as concise as possible in their narration, which can also increase the overall attraction of the report. However, based on speeding up the narrative rhythm, appropriately adding scene narration and pause narration can enrich the narrative rhythm of the entire article, improve readability, and highlight the narrative focus of the entire report through the scene and pause.

### 3.3 Analysis from the perspective of narrative structure

**Fig 8:**
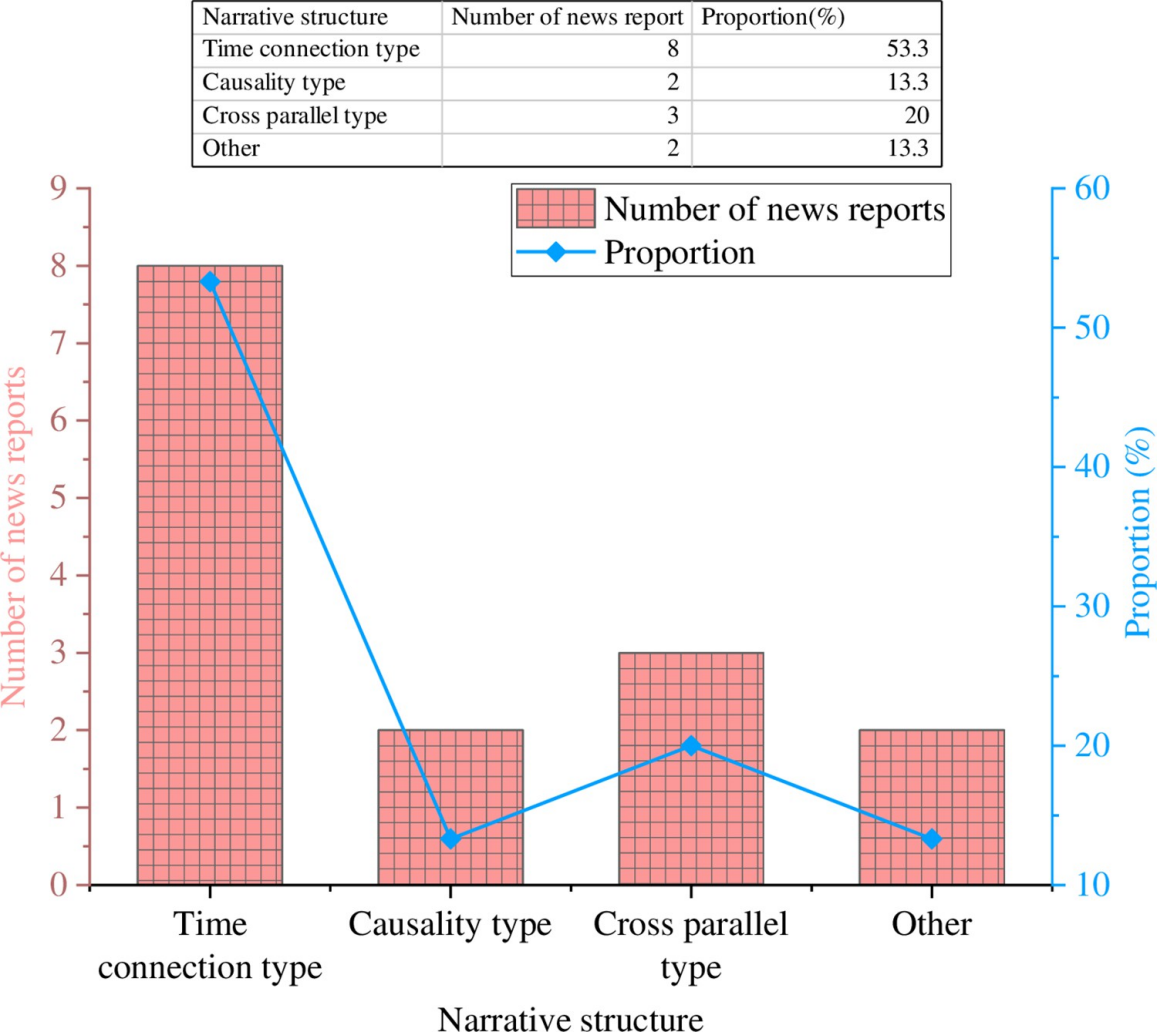
Analysis results of narrative structure.

The analysis results of the narrative structure of 15 sample news reports are denoted in Fig 8.

In Fig 8, the predominant narrative structure employed in American literary news is the time-connected narrative structure, which is utilized in 53.3% of the samples. This structure provides reporters with a well-defined conceptual framework for their writing and enables readers to swiftly comprehend and grasp the context of the event and the life course of the protagonists in the report. The second is the cross-parallel and causal narrative structure, accounting for 20% and 13.3%, respectively. The narrative structure closest to the event can restore the original state of the event to the greatest extent.

## 4. Discussion

In this research, the TF-IDF algorithm is adopted to extract pure news content from the parses of web pages, and classification algorithms such as SVM, KNN, and AdaBoost are used for comparative experiments to construct the news extraction system based on keyword features and extended DOM tree. It is found that 53.3% of the narrative structure adopts time connection mode. This narrative structure helps reporters have a clear conceptual structure when writing, helps readers to quickly grasp and understand the background of the event and the life experience of the main characters, and increases the report’s readability. In addition, regarding the study of different methods in text classification, Fesseha et al. used various CNNs to predict the pre-trained word embedding architecture of class labels. They found that CBOW-CNN using word2vec obtained the best accuracy of 93.41%, which significantly improved the accuracy of Tigrinya news classification [37]. Dhar et al. employed Bayes classification to classify documents with feature sets and found that the accuracy of naive Bayes polynomials was 98.73% [38]. Qing et al. extracted features from sentences in sentence representation, and bidirectional gated recursive units were used to access previous and next sentence features. The attention mechanism was utilized to obtain sentence representations with important word weights. In the document representation, the method used BIGRU to encode the sentence obtained in the sentence representation. Then it was decoded through the attention mechanism to get the document representation with important sentence weights [39]. The results revealed that the method was effective. In general, this research aims to achieve pure news content acquisition by using computer network technology, and to provide media practitioners with news narrative skills and strategies by analyzing the narrative characteristics and structure of American literary news. At the same time, the application and significance of this research can be further discussed in combination with the previous relevant research results.

## 5. Conclusions

To analyze the narrative characteristics of American literary news, based on the concept and characteristics of literary news, firstly, from the viewpoint of narrative characteristics of literary news, the TF-IDF algorithm is used to extract pure news content from parsed web pages. Secondly, through comparative experiments, the classification algorithms for news text classification (SVM, KNN, AdaBoost algorithm) are selected to construct a news extraction system based on keyword feature and extended DOM tree. DOM technology is utilized to parse the web page structure and extract key elements and information. The following conclusions are drawn through the analysis of 15 literary news in the American version of Mr. Fashion: (1) the most used narrative sequence in American literary news is sequence and flashback. The use of sequence can make readers understand the context of events more clearly, and make the events to be revealed gradually clear, in line with the acceptance habits of the audience. On the other hand, adding flashbacks based on order can also increase the report’s readability. (2) In terms of narrative duration, the most used ones are synopsis and ellipsis, followed by scenes and pauses. A report uses synopsis and ellipsis 15 times, 12 scenes, and 7 pauses. Based on speeding up the narrative rhythm, it enriches the narrative rhythm of the article, increases readability, and highlights the narrative focus of the whole report through scenes and pauses. (3) The most frequently used narrative structure is the time-connected narrative structure; 53.3% of the samples use this structure. This structure helps reporters to have a clear conceptual structure when writing and helps readers to quickly grasp and understand the context of events and the life course of the protagonists in the report.

The narrative structure used here is beneficial for journalists to have a clear conceptual structure when writing, helping readers quickly grasp and understand the event background and the life trajectory of the main characters in the report, and improving the readability of the report. This research deeply discusses the narrative characteristics of American literature news, and provides a reference for media practitioners in news narrative techniques and strategies. However, there are some limitations. First, the sample size is small, analyzing only 15 American literary news reports, which may not represent the whole field. Second, the research method mainly relies on keyword extraction and DOM technology to analyze the web page structure, which may have certain limitations when dealing with complex and diversified news content. In addition, the study does not discuss in depth how the findings can be applied to actual news writing and their applicability in other countries or cultural contexts. Future studies can further expand the sample size to include more different types of literary news reports to increase the representativeness and reliability of the research. Meanwhile, more advanced technologies and methods, such as natural language processing and deep learning, can be explored to improve news content extraction and classification accuracy and efficiency. Additionally, researchers can further study how to apply the obtained narrative features to actual news writing practices, and expand the scope of research to consider news narrative features in different countries and cultural contexts. These efforts will help further improve the quality of journalistic writing and the reading experience.

## Supporting information

S1 Data (ZIP)
